# Prenatal PBDEs and Neurodevelopment: Herbstman et al. Respond to Goodman et al. and to Banasik and Strosznajder

**DOI:** 10.1289/ehp.1002748R

**Published:** 2010-11

**Authors:** Julie B. Herbstman, Matthew Kurzon, Sally A. Lederman, Virginia Rauh, Deliang Tang, Frederica Perera

**Affiliations:** Columbia Center for Children’s Environmental Health, Mailman School of Public Health, Columbia University, New York, New York, Email: jh2678@columbia.edu

In our article (Herbstman et al. 2010), we reported evidence showing that children who had higher cord blood concentrations of polybrominated diphenyl ethers (PBDEs) scored lower on tests of mental and motor development at 1–4 and 6 years of age. We initiated this work based on a large body of experimental research indicating that prenatal PBDE exposure has the potential to disrupt neurodevelopment. In their letter, Banasik and Strosznajder comment that the basis for our work may be biased because of experimental design flaws in the animal studies we cited. In the introduction of our paper, we cited an extensive review article in which Costa and Giordano (2007) carefully outlined a wide variety of toxicological evidence exploring the association between prenatal PBDE exposure and neurotoxicity. The authors cited both positive and negative animal studies and also reviewed *in vitro* studies and reports outlining endocrine-disrupting effects associated with PBDE exposure. We believe that we directed *EHP* readers to sufficient evidence that provides an adequate basis for our research question. Since our manuscript was accepted for publication, an additional *in vitro* study was published; that study (Schreiber et al. 2010) demonstrated that primary fetal human neural progenitor cells exposed to BDEs 47 and 99 had decreased migration distance and reduced differentiation into neurons and oligodendrocytes. Taken together, the scientific literature provides adequate biological plausibility and raises substantial concern about the potential for PBDE-related developmental neurotoxicity in humans.

Additional comments from Goodman et al. in their letter raise the possibility that the number of samples below the limit of detection (LOD) in our study sample could be higher than in the full study population and could thereby effect the results. We explored this possibility and found that the number of study samples with PBDE levels < LOD ranged from 14% to 19% for BDE-47, 40% to 50% for BDE-99, 27% to 34% for BDE-100 and 38% to 43% for BDE-153, depending on the testing age. These are not significantly different from the proportions of samples < LOD in the full study population.

Goodman et al. also point out that PBDE concentrations in our study were measured at one point in time and were likely to change over the course of pregnancy and postnatally. It is true that little is known about changes in PBDE levels within individuals over time or the half-lives of lower brominated PBDEs in human serum. One study (Geyer et al. 2004) estimated that that the half-lives of BDEs 47, 99, 100, and 153 range from 1.8 to 6.5 years We estimated prenatal PBDE exposure based on cord blood levels at delivery. If these estimated half-lives are accurate and we assume that PBDE exposure is chronic, it is unlikely that there are substantial changes in concentrations over an approximately 9-month pregnancy. Although cord blood is adequate for assessing PBDE exposure during the prenatal and early postnatal periods, which are critical periods for neuronal differentiation and migration, we concur that it is possible that there are other windows of susceptibility that are not adequately represented by cord blood PBDE concentrations (Rice and Barone 2000). In the “Discussion” of our article, we noted the limitation that we were not able to control for postnatal PBDE exposure in our analyses.

Goodman and al. ask whether different children contribute to the observed associations at different ages and assert that most associations were found at earlier ages, suggesting that observed effects are reversible. We reported that repeated developmental test scores within an individual were correlated. We elected not to analyze the data using repeated measures because we used two different, age-appropriate neurodevelopmental tests. These tests are correlated but may not be directly comparable because they measure slightly different neurodevelopmental constructs. We disagree with the statement that associations were observed only at younger ages. Figure 1 of our article (Herbstman et al. 2010) illustrates that in our study population, the highest concentrations of exposure to prenatal PBDEs were associated with lower scores on nearly all neurodevelopmental tests at nearly all time points. Although many, but not all, of these point estimates are statistically significant, nearly all are in the same direction. Therefore, we cannot understand how this suggests that the effects of prenatal exposure are reversible.

Goodman et al. also posit that for correlated PBDEs to be causally associated with neurodevelopment, they must act via a similar mode of action (MOA). PBDE congeners are correlated, but they are of different chemical configurations and sizes. We believe that it is overly simplistic to assume that they operate via the same MOA, which is why we chose not to combine them into one exposure metric in our analyses.

Goodman et al. also raise the possibility that unmeasured confounders are a more likely explanation for the observed association between prenatal PBDEs and neurodevelopment, given that the study participants were pregnant and lived near the World Trade Center (WTC) on 11 September 2001 (9/11). Our study population consisted of women who delivered at hospitals located near the WTC; only one-fourth of our study population actually lived within 2 miles of the WTC. As we stated in our article, we cannot rule out the potential impact of unmeasured confounders. This problem is not unique to our study. Our study population is distinctive in that the participants were identified to explore the effects of prenatal exposure to the WTC after 9/11. Although we measured and controlled our analyses for many potential confounders, we cannot rule out the possibility that some unmeasured factor could be associated with both prenatal PBDE exposure and neurodevelopmental test scores and could have thereby confounded the observed associations. However, we do not understand the basis by which Goodman et al. conclude that unmeasured confounders are a more likely explanation.

Finally, Goodman et al. assert that it is impossible to determine the clinical significance of the reported associations between prenatal PBDE levels and neurodevelopmental test scores because the observed differences between exposure groups are smaller than the SD of the test instrument on a standardized population [for the Bayley Scales of Infant Development, Second Edition (BSID-II), SD = 15). We believe that Goodman et al. misinterpreted the scores derived from the Bayley scales in the context of population research. It is true that the distribution of the Mental Development Index (MDI) and Psychomotor Development Index (PDI) scores have a mean ± SD of 100 ± 15 (Bayley 1993); this is a useful guideline for interpreting the score for an individual child such that a child who scores < 1 SD of the standardized mean (score < 85) can be clinically classified as having “delayed performance.” However, the differences we noted in our article represent average difference in test scores between groups of children characterized based on their exposure levels. To illustrate our point, for BDE-100 at 36 months of age, the average test score for the highly exposed group is 6.1 points lower than the average score for the group of children with lower exposure (controlling for confounders). This shift is both statistically significant and, if confirmed, biologically relevant on a population level. Furthermore, the magnitude of this effect may be cognitively and educationally meaningful, as has been illustrated in the lead literature, where the size of the adverse effect is similar.

Our study was a well-conducted, prospective, longitudinal cohort study that demonstrated associations between prenatal PBDE exposure and adverse neurodevelopment. Given that this is the first study to report these associations in humans, we interpreted these results cautiously until they can be replicated in another study population.
